# Effects of pharmacological gap junction and sodium channel blockade on S1S2 restitution properties in Langendorff-perfused mouse hearts

**DOI:** 10.18632/oncotarget.19675

**Published:** 2017-07-28

**Authors:** Gary Tse, Tong Liu, Guangping Li, Wendy Keung, Jie Ming Yeo, Yin Wah Fiona Chan, Bryan P. Yan, Yat Sun Chan, Sunny Hei Wong, Ronald A. Li, Jichao Zhao, William K.K. Wu, Wing Tak Wong

**Affiliations:** ^1^ Department of Medicine and Therapeutics, Faculty of Medicine, Chinese University of Hong Kong, Hong Kong, China; ^2^ Li Ka Shing Institute of Health Sciences, Faculty of Medicine, Chinese University of Hong Kong, Hong Kong, China; ^3^ Tianjin Key Laboratory of Ionic-Molecular Function of Cardiovascular Disease, Department of Cardiology, Tianjin Institute of Cardiology, Second Hospital of Tianjin Medical University, Tianjin, China; ^4^ Dr. Li Dak-Sum Research Centre, The University of Hong Kong–Karolinska Institutet Collaboration in Regenerative Medicine, Hong Kong, China; ^5^ Faculty of Medicine, Imperial College London, London, UK; ^6^ School of Biological Sciences, University of Cambridge, Cambridge, UK; ^7^ Ming Wai Lau Centre for Reparative Medicine, Karolinska Institutet, Solna, Sweden; ^8^ Auckland Bioengineering Institute, The University of Auckland, Auckland, New Zealand; ^9^ Department of Anaesthesia and Intensive Care, State Key Laboratory of Digestive Disease, LKS Institute of Health Sciences, The Chinese University of Hong Kong, Hong Kong, China; ^10^ School of Life Sciences, Chinese University of Hong Kong, Hong Kong, China

**Keywords:** heptanol, conduction, repolarization, extrasystolic stimulation, S1S2 restitution

## Abstract

Gap junctions and sodium channels are the major molecular determinants of normal and abnormal electrical conduction through the myocardium, however, their exact contributions to arrhythmogenesis are unclear. We examined conduction and recovery properties of regular (S1) and extrasystolic (S2) action potentials (APs), S1S2 restitution and ventricular arrhythmogenicity using the gap junction and sodium channel inhibitor heptanol (2 mM) in Langendorff-perfused mouse hearts (n=10). Monophasic action potential recordings obtained during S1S2 pacing showed that heptanol increased the proportion of hearts showing inducible ventricular tachycardia (0/10 vs. 5/8 hearts (Fisher’s exact test, P < 0.05), prolonged activation latencies of S1 and S2 APs, thereby decreasing S2/S1 activation latency ratio (ANOVA, P < 0.05) despite prolonged ventricular effective refractory period (VERP). It did not alter S1 action potential duration at 90% repolarization (APD_90_) but prolonged S2 APD_90_ (P < 0.05), thereby increasing S2/S1 APD_90_ ratio (P < 0.05). It did not alter maximum conduction velocity (CV) restitution gradient or maximum CV reductions but decreased the restitution time constant (P < 0.05). It increased maximal APD_90_ restitution gradient (P < 0.05) without altering critical diastolic interval or maximum APD_90_ reductions. Pro-arrhythmic effects of 2 mM heptanol are explicable by delayed conduction and abnormal electrical restitution. We concluded that gap junctions modulated via heptanol (0.05 mM) increased arrhythmogenicity through a delay in conduction, while sodium channel inhibition by a higher concentration of heptanol (2 mM) increased arrhythmogenicity via additional mechanisms, such as abnormalities in APDs and CV restitution.

## INTRODUCTION

Gap junctions and sodium channels are the major molecular determinants of conduction velocity (CV) of action potentials (APs) travelling through the myocardium [1–4]. Heptanol is a pharmacological agent that uncouples gap junctions at concentrations < 2 mM and additionally inhibits sodium channels > 2 mM [5]. A number of investigators have examined the effects of this agent on ventricular arrhythmogenicity, demonstrating different effects in various model systems [6–9]. The reasons are likely attributable to distinct electrophysiological mechanisms observed in different animal models, pathophysiological conditions and drug concentrations used. For example, Callans and his colleagues demonstrated in a canine myocardial infarction model, that heptanol had a bimodal effect on ventricular arrhythmogenicity, with 0.5 mM heptanol increasing, and 1 mM heptanol decreasing, the incidence of induced VT [6]. In rabbit preparations, 1 mM heptanol reduced CV, increased the excitable gap and to a lesser extent the effective refractory period (ERP) as well as prolonged the cycle length during ventricular tachycardia [7].

By contrast, experiments from our group demonstrated ventricular pro-arrhythmic effects of heptanol at both 0.05 and 2 mM in Langendorff-perfused mouse hearts [9]. We showed that heptanol at 0.05 mM reduced CV without altering ERP or action potential duration (APD), leading to a reduction in excitation wavelength (λ = CV x ERP). At a higher concentration of 2 mM, in addition to decreased CV, ERP was also increased, but this also led to a decrease in λ. However, none of the experiments described above explicitly examined the contributions of the conduction or repolarization properties of the extrasystolic APs that serve to initiate ventricular arrhythmias, nor did they examine restitution properties thought to be important in the generation of local tissue electrophysiological heterogeneities.

Firstly, conduction abnormalities of extrasystolic APs can increase arrhythmia inducibility [10]. Secondly, altered gap junction or sodium function can influence electrical restitution [11], potentially initiating APD alternans through steep APD restitution [12]. Alternans that are spatially discordant are thought to be more arrhythmogenic than those that are concordant. Spatially concordant can be converted to discordant APD alternans by mechanisms such as abnormal CV restitution [13–16].

In this study, therefore, we tested the hypotheses that i) an increased ratio of the activation latency of the extrasystolic AP to that of the regular AP, reflecting slower CV, and ii) altered ratio of APD of the extrasystolic AP to that of the regular AP and iii) abnormal CV or APD restitution contribute to the heptanol-induced arrhythmogenesis.

## RESULTS

Ventricular arrhythmogenicity and its relationship to action potential activation and recovery properties of the regular and extrasystolic action potentials (APs) were examined before and after introduction of 0.05 or 2 mM heptanol in Langendorff-perfused mouse hearts. The right ventricular epicardium was electrically stimulated using a regular 8 Hz or S1S2 pacing protocol. Monophasic action potential (MAP) waveforms were recorded from the left ventricular epicardium. Ventricular tachycardia (VT) was defined as a series of five or more action potentials with coupling intervals closer than the basic cycle length (BCL).

### Arrhythmogenicity studies and electrophysiological properties of S1 and S2 action potentials in the presence or absence of heptanol during S1S2 pacing

**Figure 1:**
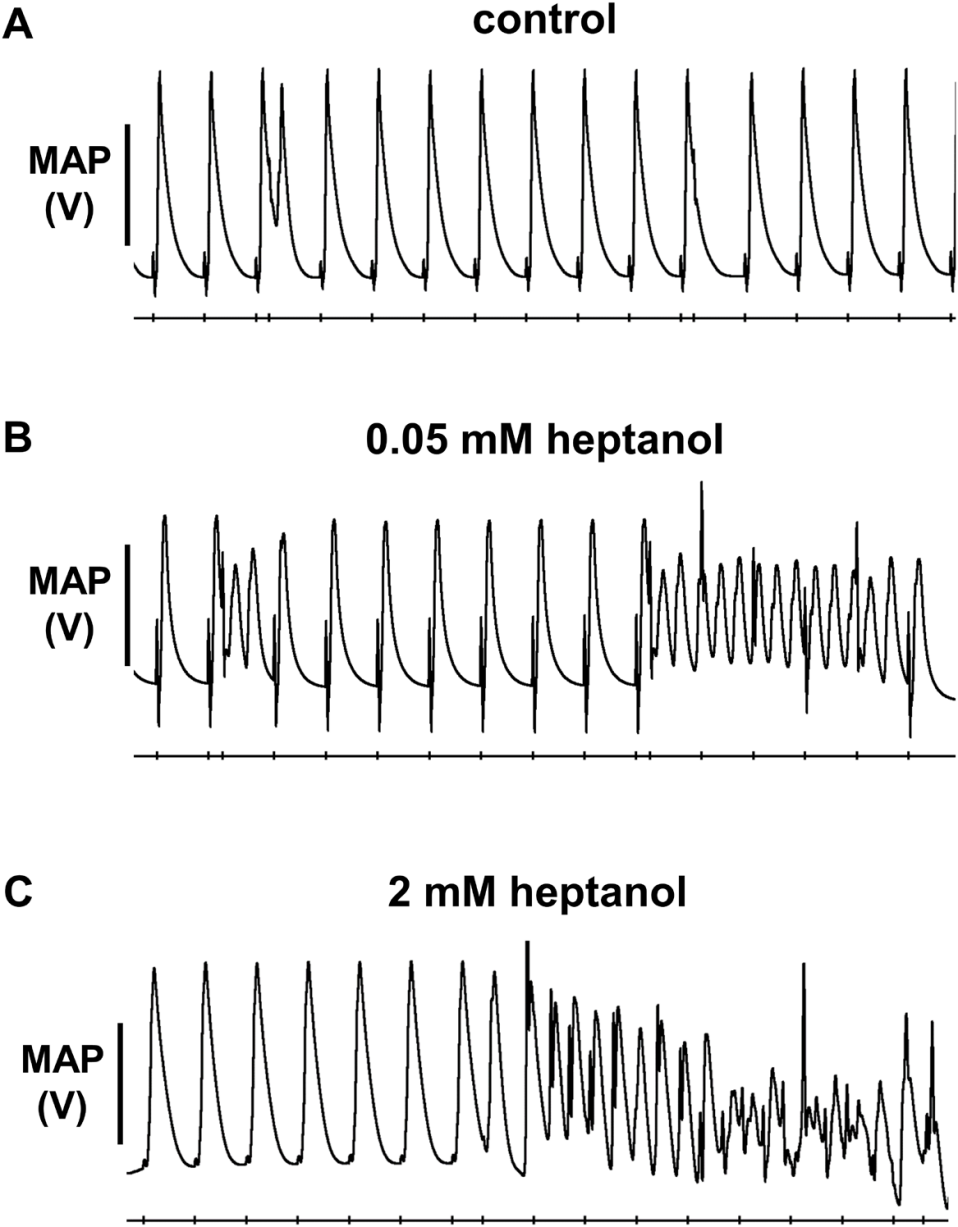
No ventricular arrhythmias were observed before introduction of heptanol **(A),** in contrast to inducible ventricular tachycardia (VT) occurring after 0.05 mM **(B)** or 2 mM **(C)** heptanol treatment during S1S2 pacing.

**Figure 2:**
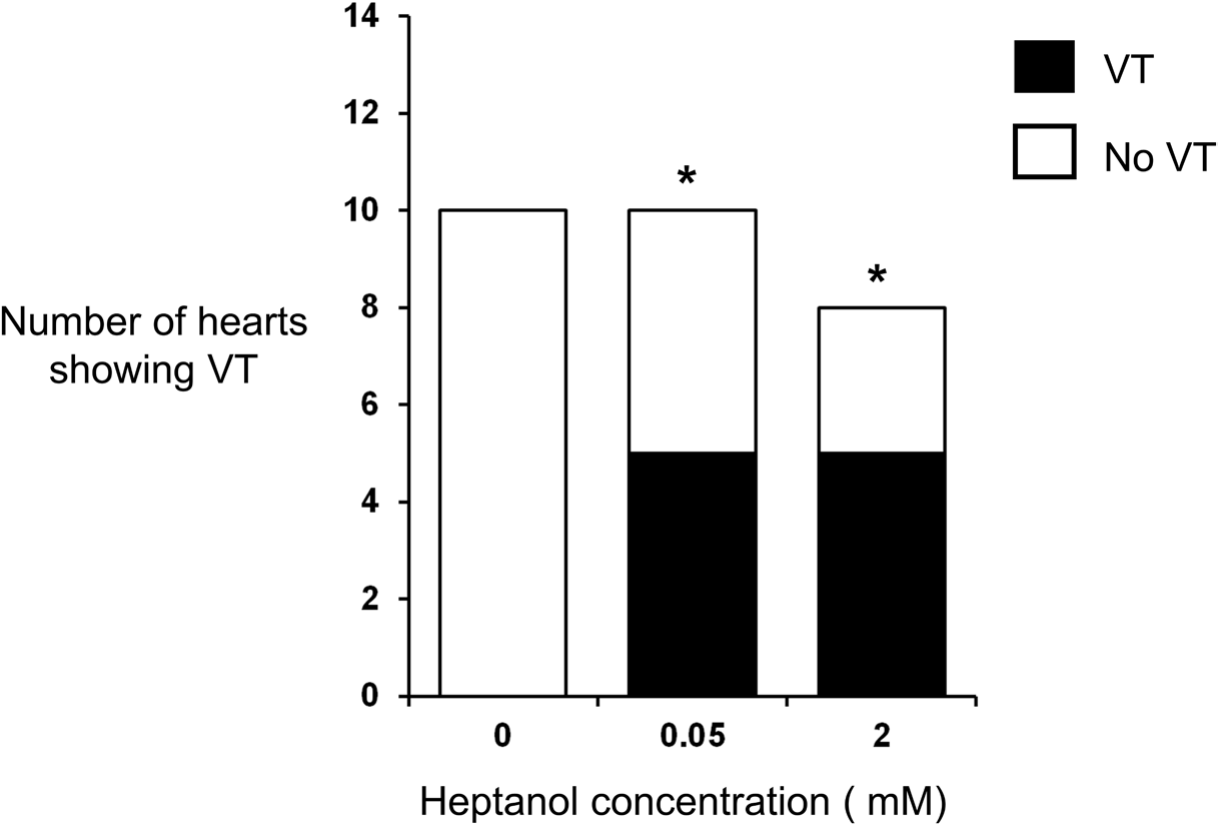
Incidence of inducible ventricular tachycardia (VT) under control conditions and in the presence of 0.05 mM or 2 mM heptanol

**Figure 3:**
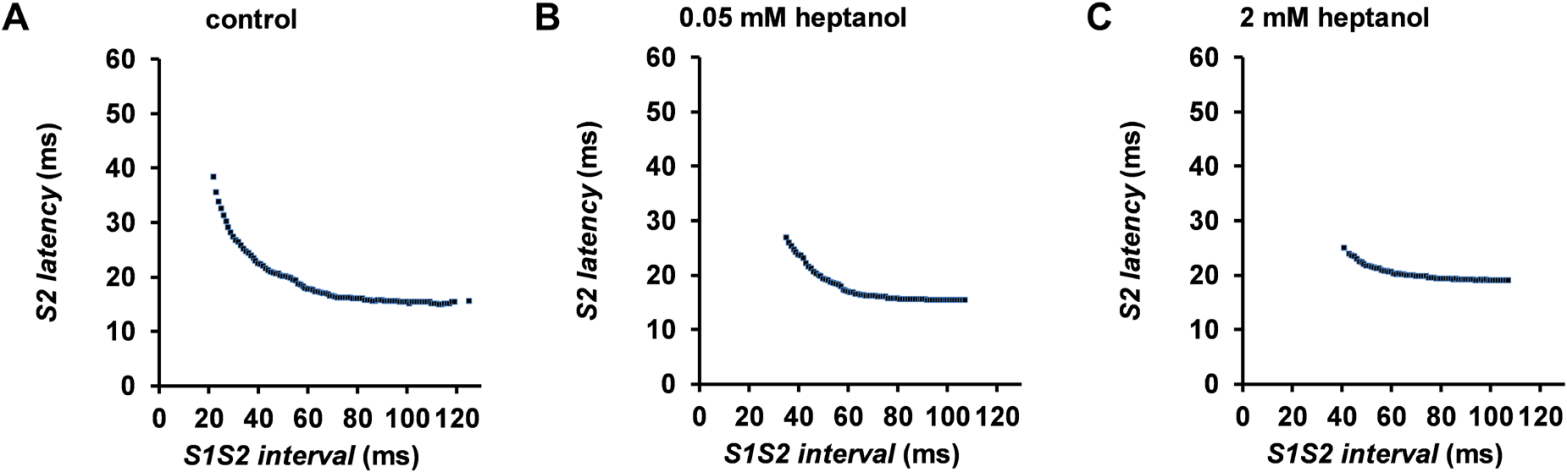
S2 activation latency plotted against S1S2 interval before **(A)** and after introduction of 0.05 mM **(B)** or 2 mM **(C)** heptanol from a representative heart.

**Figure 4:**
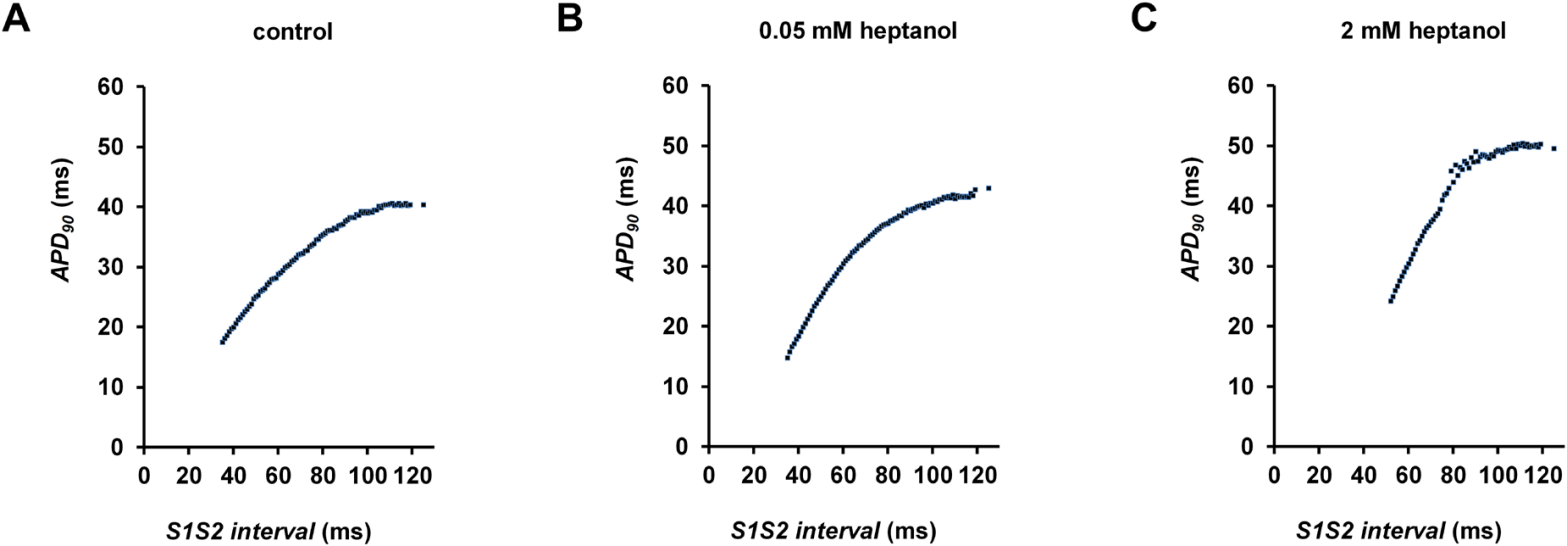
S2 APD_90_ plotted against S1S2 interval before **(A)** and after introduction of 0.05 mM **(B)** or 2 mM **(C)** heptanol.

The initial experiments confirmed previous findings that heptanol at both 0.05 and 2 mM exerted ventricular pro-arrhythmic effects, as demonstrated by the presence of inducible VT during the S1S2 protocol (Figure 1A to 1C). Under control conditions, 10 out of 10 hearts reached refractory outcomes during programmed electrical stimulation. In the presence of 0.05 mM heptanol, this proportion was increased to 5 out of 10 hearts. At a higher concentration of 2 mM heptanol, 5 out of 8 hearts showed evidence of inducible VT. Therefore, heptanol at both concentrations exerted significant arrhythmogenic effects compared with control (Figure 2; Fisher’s Exact Test, P < 0.05). The S2 activation latency increased (Figure 3A to 3C) with progressive shortening in the S1S2 interval both before and after introduction of 0.05 or 2 mM heptanol. Similarly, the S2 action potential duration (APD at 90% repolarization, APD_90_) decreased with a progressive shortening in the S1S2 interval under these pharmacological conditions (Figure 4A to 4C).

**Figure 5:**
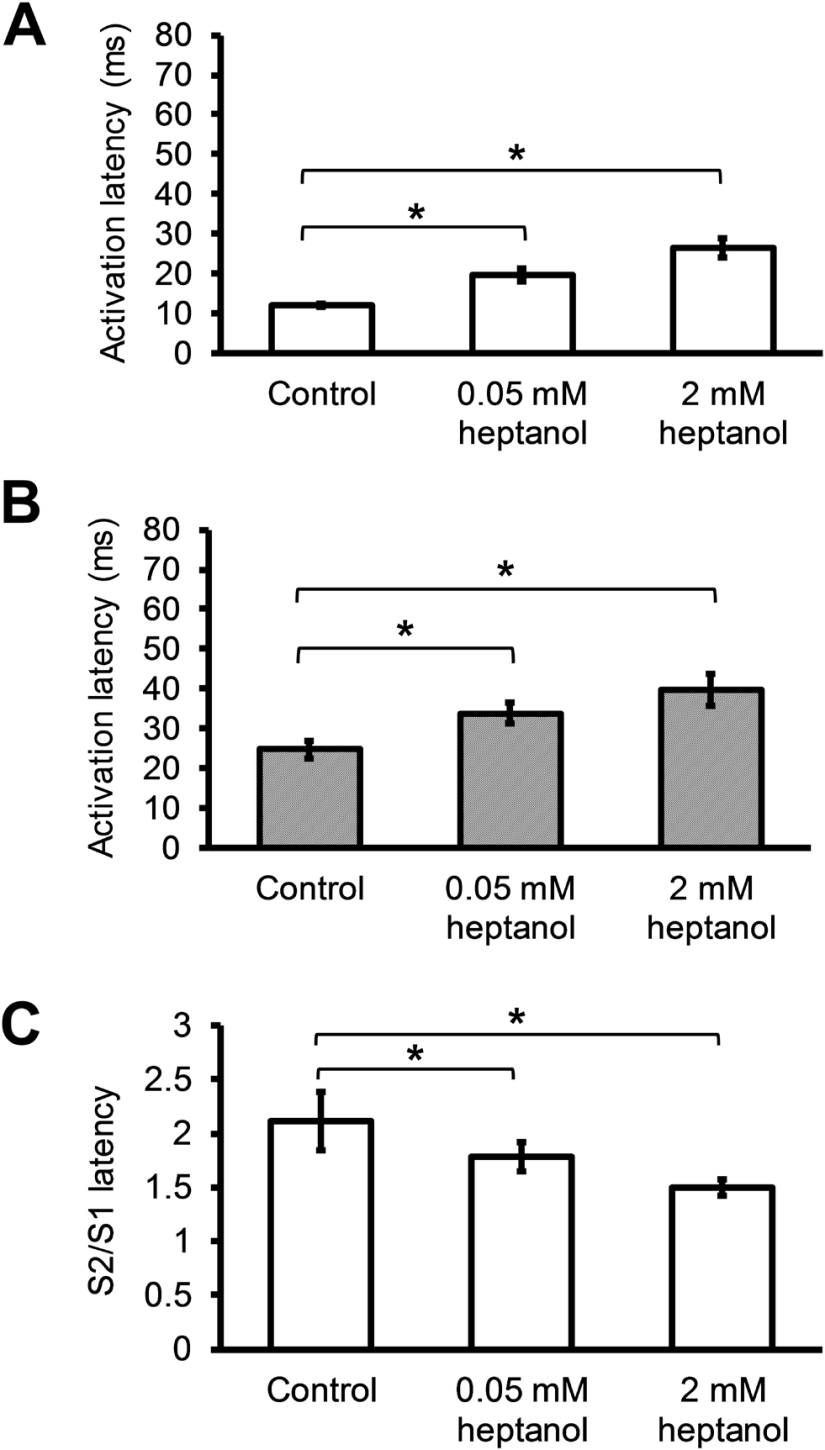
S1 activation latencies **(A)** and S2 activation latencies **(B)** immediately before reaching a refractory or an arrhythmic outcome, and S2 latency / S1 latency ratio before and after introduction of 0.05 mM or 2 mM heptanol **(C)**.

Activation latency of the regular (S1) action potential (AP) was increased from 12.0 ± 0.4 to 19.7 ± 1.8 ms by 0.05 mM heptanol (n = 10), and increased further to 26.5 ± 2.4 ms by 2 mM heptanol (n = 8) (Figure 5A; *P* < 0.05). Similarly, activation latency of the extrasystolic (S2) AP was increased from 24.7 ± 2.2 to 33.9 ± 2.6 ms by 0.05 mM heptanol (n = 10), and further increased to 39.6 ± 4.0 ms by 2 mM heptanol (n = 8) (Figure 5B; *P* < 0.05). Consequently, the S2 to S1 activation latency ratio was decreased from 2.1 ± 0.3 to 1.8 ± 0.1 by 0.05 mM heptanol (n = 10), and further to 1.5 ± 0.1 ms by 2 mM heptanol (n = 8) (Figure 5C).

**Figure 6:**
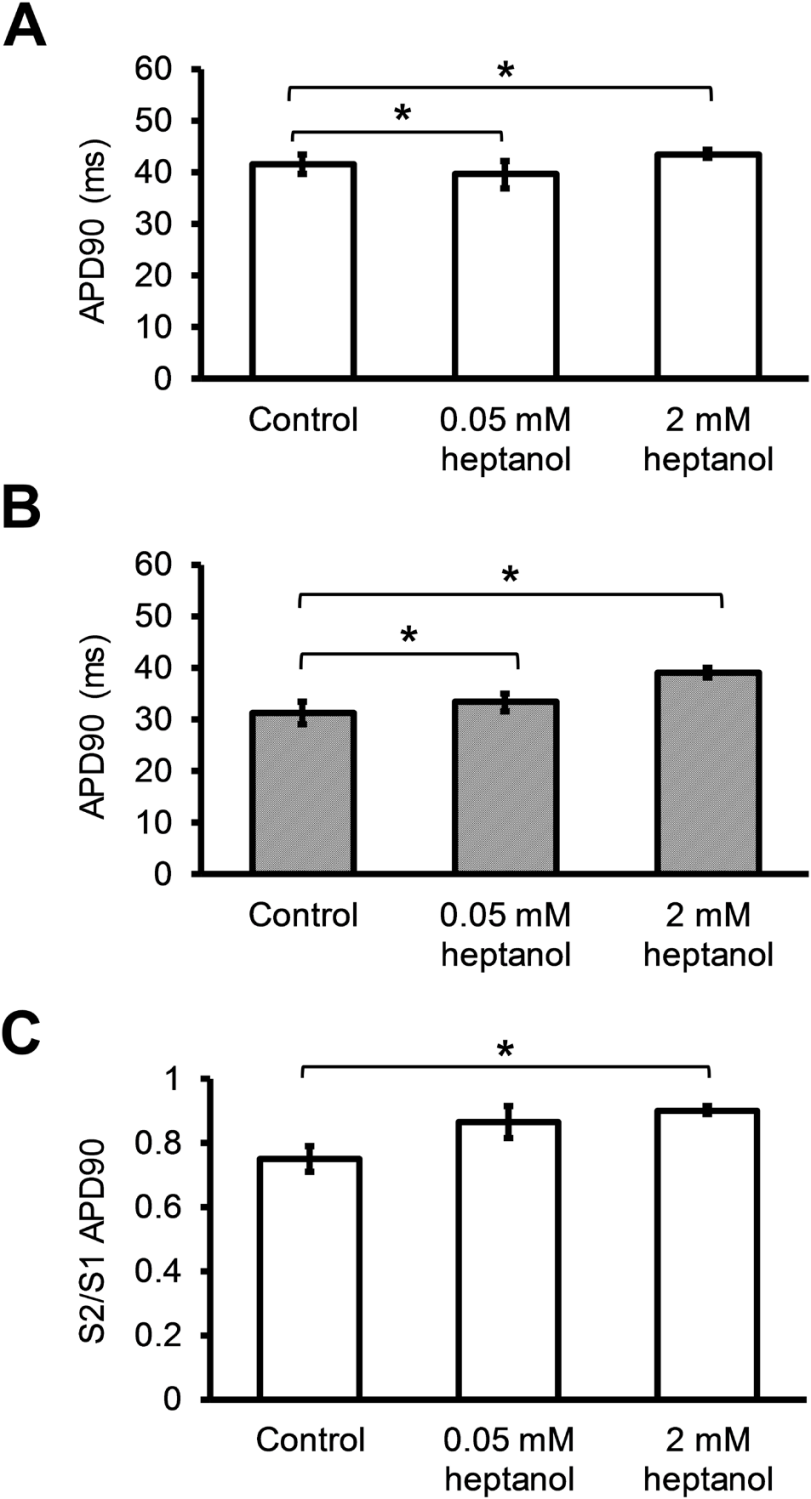
S1 APD **(A)** and S2 APD **(B)** immediately before reaching a refractory or an arrhythmic outcome and S2 APD_90_ / S1 APD_90_ ratio before and after introduction of 0.05 mM or 2 mM heptanol **(C)**.

**Figure 7:**
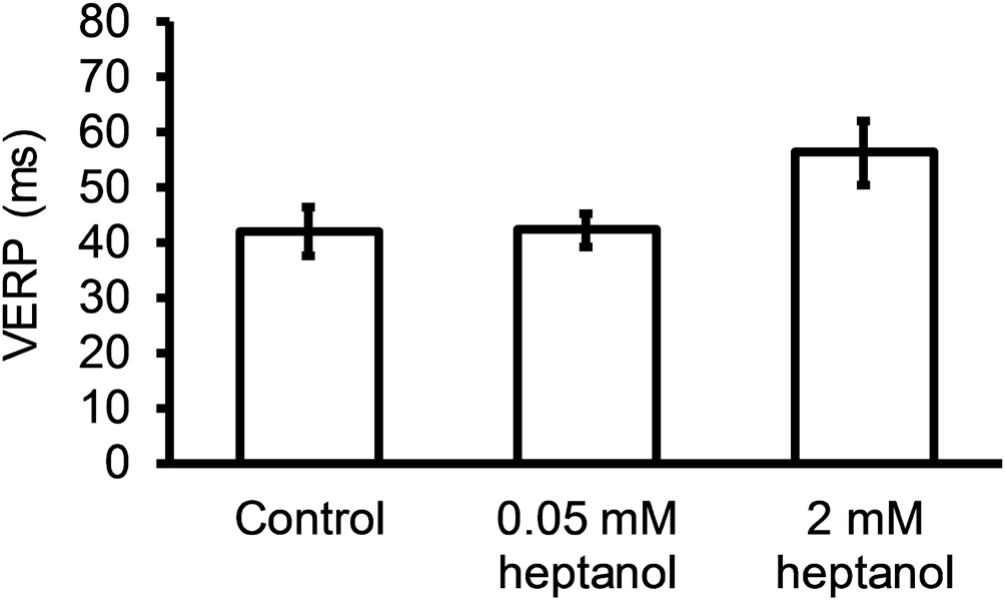
Ventricular effective refractory period (VERP) before and after introduction of 0.05 mM or 2 mM heptanol

APD_90_ of the S1 AP was not significantly altered by 0.05 mM or 2 mM heptanol (Figure 6A; 41.6 ± 1.9 vs 39.5 ± 2.7 vs 43.3 ± 0.8 ms; *P* > 0.05). Whilst APD_90_ of the S2 AP was similarly unaltered by 0.05 mM heptanol (n = 10) (Figure 6B; 31.1 ± 2.2 vs. 33.3 ± 1.7; *P* > 0.05), it was prolonged by 2 mM heptanol to 39.1 ± 1.0 (n = 8) (*P* < 0.05). The S2 to S1 APD_90_ ratio was consequently unaltered by 0.05 mM heptanol (n = 10) (Figure 6C; 0.75 ± 0.04 vs. 0.86 ± 0.05; *P* > 0.05), but reduced by 2 mM heptanol to 0.90 ± 0.01 (n = 8) (*P* < 0.05). VERP was not altered by 0.05 mM heptanol (n = 10) (42.0 ± 4.5 ms vs. 42.2 ± 3.0 ms; P > 0.05) but was increased by 2 mM heptanol (n = 8) (Figure 7; 56.3 ± 5.9 ms; P < 0.05).

### APD_90_ and CV restitution properties determined from S1S2 pacing in the presence or absence of heptanol

**Figure 8:**
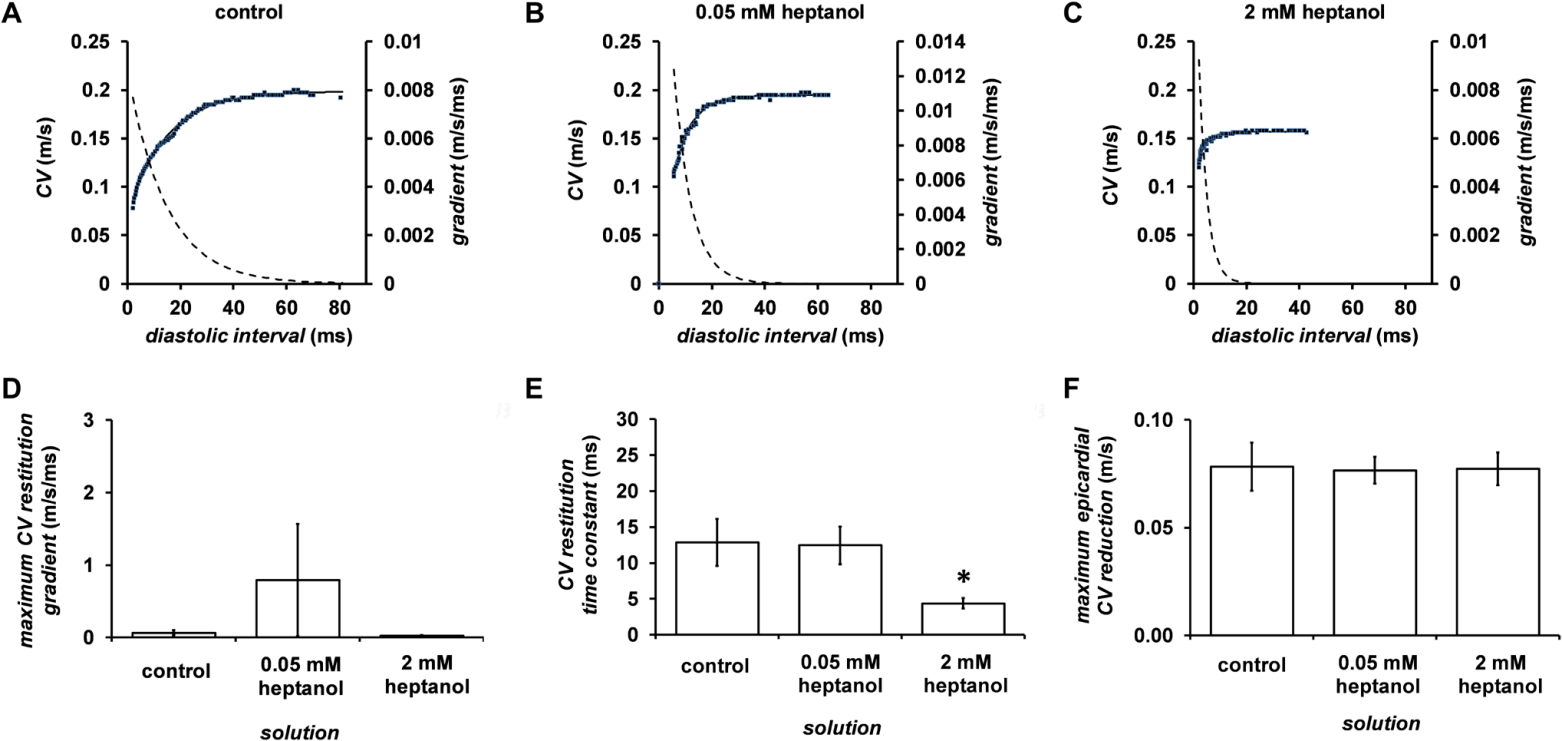
Restitution curves plotting CV against preceding DI obtained before **(A)** and after introduction of 0.05 **(B)** or 2 mM heptanol **(C)**. Curves were fitted with mono-exponential growth functions obtained by least-squares fitting to the values of CV and DI (*solid lines*, left ordinates). Gradients were obtained by differentiation of the fitted functions (*broken lines*, right axes). Maximum CV restitution gradients **(D)**, time constants of restitution curves **(E)** and maximum CV reductions **(F)**.

Figures 8A to 8C show examples of conduction velocity (CV) restitution curves (*solid lines*, left ordinates) and their gradients (*broken lines*, right axes) under the same pharmacological conditions described above, with fitted parameters summarized in Table 1. Maximum CV restitution gradients (Figure 8D) or maximum CV reduction (Figure 8F) was not altered by 0.05 (n = 10) or 2 mM heptanol (n = 8). By contrast, the time constants τ of the restitution curves was unaltered by 0.05 mM heptanol (n = 10) but was decreased by 2 mM heptanol (n = 8) (Figure 8E).

**Table 1:** Fitted parameters for CV restitution curves

Condition	y_o_ (m/s)			A (m/s)			τ (s)		
control	0.216	±	0.013	-0.255	±	0.092	0.0129	±	0.003
0.05 mM heptanol	0.180	±	0.012	-1.350	±	1.215	0.0124	±	0.003
2 mM heptanol	0.134	±	0.008	-0.102	±	0.027	0.0044	±	0.001

**Figure 9:**
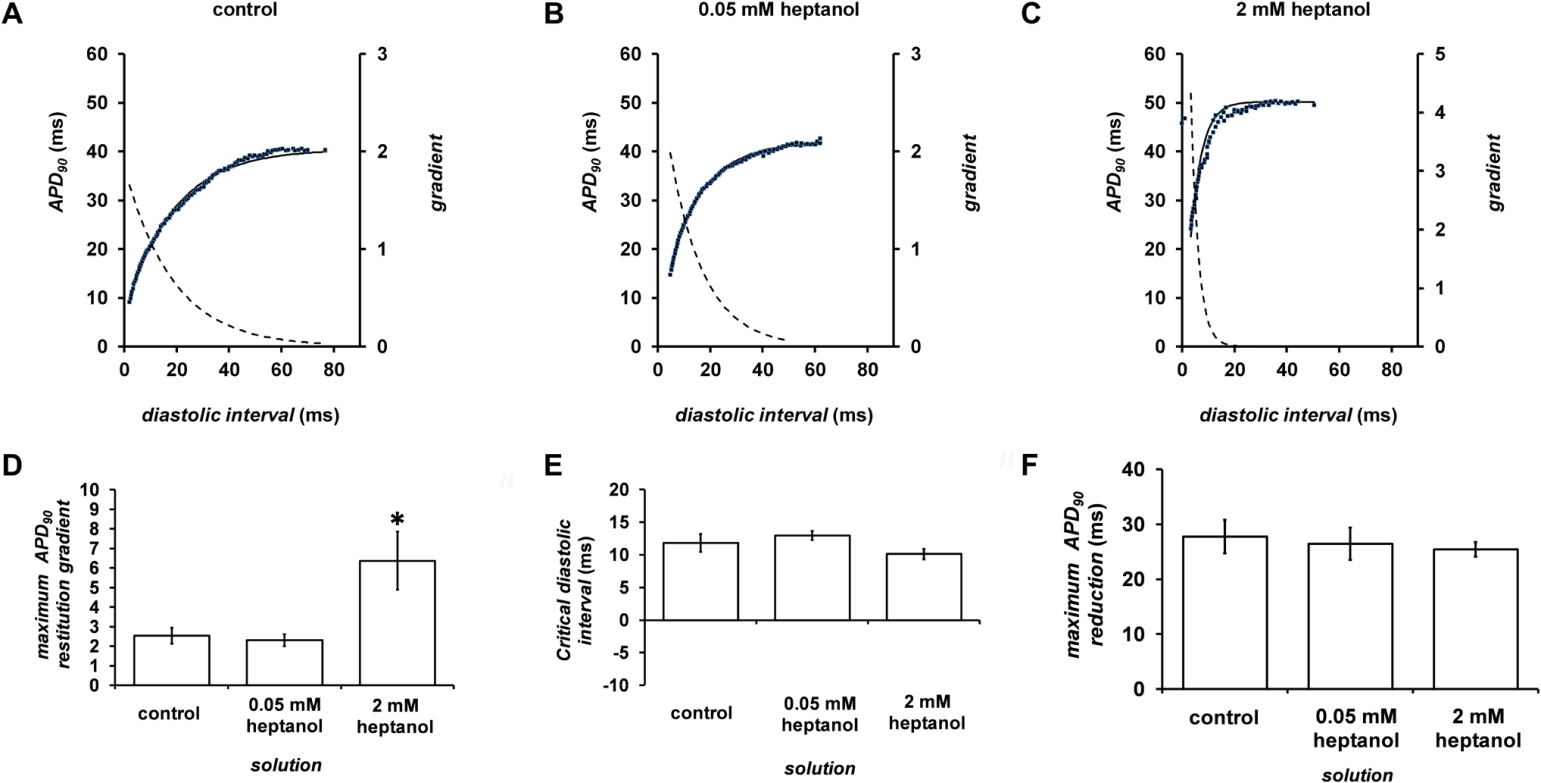
Restitution curves plotting APD_90_ against preceding diastolic interval (DI) before **(A)** and after introduction of 0.05 **(B)** or 2 mM heptanol **(C)**. Curves were fitted with mono-exponential growth functions obtained by least-squares fitting to the values of APD_90_ and DI (*solid lines*, left ordinates). Gradients were obtained by differentiation of the fitted functions (*broken lines*, right axes). Maximum APD_90_ restitution gradients **(D)**, critical diastolic intervals **(E)** and maximum APD_90_ reductions **(F)**.

Figure 9A to 9C shows examples of APD_90_ restitution curves (*solid lines*, left ordinates) and their gradients (*broken lines*, right axes) before or after introduction of 0.05 (n = 10) or 2 mM heptanol (n = 8); fitted parameters are summarized in Table 2. Maximal APD_90_ restitution gradients was not altered by 0.05 mM heptanol (n = 10) but was increased by 2 mM heptanol (n = 8) (Figure 9D). By contrast, both DI_crit_ (Figure 9E) and maximum APD_90_ reductions (Figure 9F) was unaltered by 0.05 (n = 10) or 2 mM heptanol (n = 8) (*P* < 0.05).

**Table 2:** Fitted parameters for APD restitution curves

Condition	y_o_ (s)			A (s)			τ (s)		
control	0.039	±	0.002	-0.045	±	0.010	0.011	±	0.001
0.05 mM heptanol	0.043	±	0.002	-0.038	±	0.002	0.012	±	0.001
2 mM heptanol	0.051	±	0.002	-0.068	±	0.017	0.004	±	0.001

## DISCUSSION

Extrasystolic action potentials can initiate ventricular tachy-arrhythmias, which can be sustained in the presence of favourable re-entrant substrates such as areas of abnormal conduction [17–19]. A key determinant of cardiac conduction is gap junctions [20–22], whose roles in ventricular arrhythmogenesis have been extensively studied in different animal systems [23] such as canine myocardial infraction model [24] and rabbit heart failure model [25]. These models are excellent for characterization of long-term electrophysiological and structural remodeling of the myocardium.

Of the different animal models, mouse hearts have been a popular system for investigating cardiac electrophysiology owing to their ease of reproduction, access, genetic and pharmacological manipulation [26, 27]. For example, the effects of loss of the gap junction protein, connexin 43, have been studied in great detail [13, 22, 28–36]. Genetic modification has been achieved by cardiac-restricted inactivation of Cx43 and subsequent crossing with Cre recombinase produced mice with mosaicism [28]. In this model, Cx43 levels were reduced by 86 to 95%. Moreover, in heterozygous Cx43^+/-^ mice, Cx43 expression was decreased by 45 to 50%. CV was unaffected [13, 22, 30, 31, 34, 35] or decreased up to 44% in these models [29, 32, 33].

In addition to these genetic models, pharmacological studies have tested the acute effects of altered gap junction function using uncouplers such as carbenoxolone [37], palmitoleic acid [38] or heptanol [6, 7] have been used. Of these agents, heptanol uncouples at concentrations < 2 mM and additionally inhibits sodium channels when applied at concentrations > 2 mM [5, 39, 40]. In infarcted canine hearts, heptanol had a dual effect on ventricular arrhythmogenicity, increasing ventricular arrhythmogenicity at 0.5 mM but decreasing arrhythmogenicity at 1 mM [6]. By contrast, in mouse hearts, pro-arrhythmic effects have been observed across the concentrations from 0.05 mM to 2 mM [9, 41].

Thus, in the presence of 0.05 mM heptanol, an increase in the incidence of inducible, but not spontaneous, ventricular tachycardia (VT) was observed, which was associated with increases in activation latencies, which reflect reduced conduction velocity or alterations of the conduction pathway, in an absence of alterations in action potential durations (APDs) or ventricular effective refractory periods (VERPs) [9]. By contrast, at a higher concentration of 2 mM, both the incidences of spontaneous and inducible VT were increased [41]. These arrhythmogenic phenomena were associated with further increases in activation latency and VERP. Therefore, heptanol at 2 mM, but not 0.05 mM, induced post-repolarization refractoriness, which is expected to inhibit, rather than induce, ventricular arrhythmogenesis.

However, in these previous studies, neither the behaviour of hearts once these extrasystolic APs were initiated, nor abnormal electrical restitution, was investigated. Therefore, the present study examined the effects of heptanol on conduction slowing and abnormal repolarization of extrasystolic APs as well as abnormal electrical restitution, and their relationships with ventricular arrhythmogenicity. Our initial experiments first confirmed the pro-arrhythmic effects of heptanol at both 0.05 and 2 mM using programmed electrical stimulation that delivered increasingly premature extrasystolic, S2 pacing stimuli following trains of regular S1 stimuli. Heptanol increased the activation latency of both the S1 and S2 APs. However, it produced a decrease, rather than increase, in the S2 to S1 activation latency ratio. In other words, there was a smaller degree of conduction slowing or change in conduction pathway of extrasystolic APs relative to the regular APs in the presence of heptanol. Therefore, the arrhythmogenesis observed was not explicable by conduction defects of the extrasystolic APs. Moreover, previous experiments found that arrhythmic outcomes were associated with lower CVs in their initiating extrasystolic APs than refractory outcomes in a long QT syndrome mouse model [10]. However, activation latencies in the arrhythmic and refractory groups were not significantly different from each other in our model.

Moreover, previous reports have associated increased arrhythmogenicity with increases in maximum APD_90_ restitution gradients, critical diastolic intervals (DIs, DI_crit_), and APD_90_ heterogeneity in a pharmacological mouse model of long QT syndrome [42, 43]. Increased arrhythmogenicity in other model systems has been associated with abnormal activation latency restitution properties. The latter is observed as increased maximum restitution gradients [14], increased time constants of the restitution curves [15, 44] and increased heterogeneity in activating latency, given by maximal increase between the longest and shortest S1S2 intervals studied was seen in D600-treated rabbit hearts [12]. Restitution analysis of data obtained during PES revealed a steeper APD restitution and a shorter time constant for the CV restitution curve in the presence of 2 mM heptanol. At a lower concentration of 0.05 mM, all of the APD and CV restitution parameters remained unaltered.

### Limitations

There are several limitations of this study, which are mainly due to the experimental methodology used. Firstly, it was not possible to determine the exact conduction velocities (CVs) as single point measurements were made from the hearts. Altered activation latencies in this study could well be attributed to alterations in conduction path of the propagating action potentials in addition to reduced conduction velocity. A better method would be the use of optical mapping, which can determine CVs from multi-point recordings, and distinguish reduced CVs from alterations in conduction path as well as orientation of myocardial fibres. Secondly, higher concentrations of heptanol (2 mM in this study) have dual effects on activation and repolarization. Therefore, it was not possible to elucidate what selective sodium channel block, gap junctional block or repolarization heterogeneity plays in heptanol-induced arrhythmogenesis. Future studies using selective gap junction blockers and openers would provide additional insight.

## CONCLUSIONS

In conclusion, our data suggest that gap junctions modulated via heptanol (0.05 mM) increased arrhythmogenicity through a delay in conduction, while sodium channel inhibition by a higher concentration of heptanol (2 mM) increased arrhythmogenicity by additional mechanisms, such as abnormalities in APDs and CV restitution.

## MATERIALS AND METHODS

### Solutions

Krebs-Henseleit solution (composition in mM: NaCl 119, NaHCO_3_ 25, KCl 4, KH_2_PO_4_ 1.2, MgCl_2_ 1, CaCl_2_ 1.8, glucose 10 and sodium pyruvate 2, pH 7.4), which has been bicarbonate-buffered and bubbled with 95% O_2_–5% CO_2_, was used in the experiments described in this study. Heptanol (Sigma, Dorset, UK; density: 0.82 g ml) is an agent that remains soluble in aqueous solutions up to 9 mM (The Merck Index, New Jersey, USA). Krebs-Henseleit solution was used to dilute the heptanol solution to produce a final concentration of 0.05 and 2 mM.

### Preparation of Langendorff-perfused mouse hearts

This study was approved by the Animal Welfare and Ethical Review Body at the University of Cambridge. Wild-type mice of 129 genetic background between 5 and 7 months of age were used. They were maintained at room temperature (21 ± 1°C) and were subjected to a 12:12 h light / dark cycle with free access to sterile rodent chow and water in an animal facility. Mice were terminated by dislocation of the cervical spine in accordance with Sections 1(c) and 2 of Schedule 1 of the UK Animals (Scientific Procedures) Act 1986. After removal from their chest cavities, the hearts were submerged in ice-cold Krebs-Henseleit solution. The aortas were cannulated using a custom-made 21-gauge cannula prefilled with ice-cold buffer. A micro-aneurysm clip (Harvard Apparatus, UK) was used to secure the hearts onto the Langendorff perfusion system. Retrograde perfusion was carried out at a flow rate of 2 to 2.5 ml min^-1^ by use of a peristaltic pump (Watson–Marlow Bredel pumps model 505S, Falmouth, Cornwall, UK). The perfusate passed through successively 200 and 5 μm filters and warmed to 37°C using a water jacket and circulator before arriving at the aorta. Approximately 90% of the hearts regained their pink colour and spontaneous rhythmic activity. These were therefore studied further. The remaining 10% did not and were discarded. The hearts were perfused for a further 20 minutes to minimise residual effects of endogenous catecholamine release, before their electrophysiology properties were characterized.

### Stimulation protocols

Paired platinum electrodes (1 mm interpole distance) were used to stimulate the right ventricular epicardium electrically. This took place at 8 Hz, using square wave pulses of 2 ms in duration, with a stimulation voltage set to thrice the diastolic threshold (Grass S48 Stimulator, Grass-Telefactor, Slough, UK) immediately after the start of perfusion. The S1S2 protocol was used to assess arrhythmogenicity and identify re-entrant substrates. This consisted of a drive train of eight regularly paced S1 stimuli separated by a 125 ms basic cycle length (BCL), followed by premature S2 extra-stimuli every ninth stimulus. The S1S2 interval was first set to 125 ms and then successively reduced by 1 ms with each nine stimulus cycle until arrhythmic activity was initiated or refractoriness was reached, whereupon the S2 stimulus elicited no ventricular response.

### Recording procedures

A Monophasic action potential (MAP) electrode was used to record MAPs from the left ventricular epicardium (Linton Instruments, Harvard Apparatus). The stimulating and recording electrodes were maintained at constant positions separated approximately by distance of 3 mm. This means the inverse of the activation latencies is proportional to the conduction velocity (CV). All recordings were performed using a baseline cycle length (BCL) of 125 ms (8 Hz) to exclude rate-dependent differences in action potential durations (APDs). MAPs were pre-amplified using a NL100AK head stage, amplified with a NL 104A amplifier and band pass filtered between 0.5 Hz and 1 kHz using a NL125/6 filter (Neurolog, Hertfordshire, UK) and then digitized (1401plus MKII, Cambridge Electronic Design, Cambridge, UK) at 5 kHz. Waveforms were analysed using Spike2 software (Cambridge Electronic Design, UK). MAP waveforms that did not match established criteria for MAP signals were rejected [45, 46]. They must have “stable baselines, fast upstrokes, with no inflections or negative spikes, and a rapid first phase of repolarization”. 0% repolarization was measured at the peak of the MAP and 100% repolarization was measured at the point of return of the potential to baseline [45, 47, 48].

The following parameters were obtained from the experimental records: (1) Activation latency, defined as the time difference between the stimulus and the peak of the MAP, of the action potentials obtained follow S1 and S2 stimulation at different S1S2 intervals; (2) APD_x_, the time difference between the peak of the MAP and x = 30, 50, 70 and 90% repolarization of the S1 APs and S2 APs at different S1S2 intervals; (3) restitution gradient obtained from restitution curves plotting conduction velocity (CV, inter-electrode distance / activation latency) against the previous DI, assuming its maximal value at the shortest S1S2 interval studied; (4) CV restitution curve time constant, τ; (5) Maximum CV reduction, a measure of restitution heterogeneity, defined as the maximum change in CV observed between the longest and shortest S1S2 interval achieved during PES [12, 49]; (6) APD_90_ restitution gradient obtained from restitution curves plotting APD_90_ against the previous diastolic interval (DI), assuming a maximum gradient at the shortest S1S2 interval studied; (7) Critical DI, DI_crit_, defined as the DI at which the gradient of the APD_90_ restitution curve reaches unity; (8) Maximum APD_90_ reduction, a measure of APD_90_ restitution heterogeneity, defined as the maximum APD_90_ reduction observed between the longest and shortest S1S2 intervals achieved during PES [12, 50];

In this study, restitution curves were constructed using the PES data obtained above, by plotting activation latency or APD_90_ against the preceding DI, and were then fitted with an exponential function of the form y=y0+Ae−xτ by a least-squares method using a Levenberg-Marquardt algorithm. *y* represents either APD_90_ or CV, and *x* represents DI, whereas y_0_, A and τ are constants. The gradient is given by dydx = Aτe−xτ, assuming its maximal value at the shortest S1S2 interval reached during PES. DI_crit_ was defined as the DI at which the gradient of the fitted function reached unity. Maximum CV or maximum APD_90_ reduction, reflecting heterogeneity in restitution, was defined as the difference between values obtained at the longest S1S2 interval and those obtained at the shortest S1S2 interval.

### Statistical analysis

All values were expressed as mean ± standard error of the mean (SEM). Categorical data were compared with Fisher's exact test (two-tailed). Numerical data were compared by one-way analysis of variance (ANOVA). *P* < 0.05 was considered statistically significant and was denoted by * in the figures.

## Abbreviations

APD: action potential duration
CV: conduction velocity
DI_crit_: critical diastolic interval
ERP: effective refractory period
VERP: ventricular effective refractory period
